# Supplementary material for: Patterns of Intron Gain and Loss in Fungi
Source: PLoS Biol. 2004 Nov 30;2(12):e422. doi: 10.1371/journal.pbio.0020422 (PMC532390; doi:10.1371/journal.pbio.0020422)
Supplement: Table S1 — Also available at http://genes.mit.edu/NielsenEtAl/. (4.3 MB ZIP). [file pbio.0020422.st001.zip › NielsenEtAl/html/398.html]

AN6250.1.NCU01803.1.MG06681.1.FG10878.1


```
 CLUSTAL W (1.82) Multiple Sequence Alignments - Introns Inserted


Sequence 1: NCU01803.1	437 aa
Sequence 2: MG06681.1	381 aa
Sequence 3: FG10878.1	358 aa
Sequence 4: AN6250.1	403 aa
Alignment Length: 459 aa
Number Identitical Residues: 86 aa
Alignment Score (without introns) 6002


MG06681.1 	MHQT--------------------~------------------VD-----~--IVMDKTI
NCU01803.1	MYNTNSMQTPDDAEAPLSFIFPIG1YSYYATSKSLSTHLYQKLVDRYWRR~SGTLLYRPN
FG10878.1 	------------------------~-------------------------~----MYRPD
AN6250.1  	MSSQVTNATRADIAN---------~-----------QIMAVSRRSPTPQP2SGTLYYKQN
          	  .  :  :  .                             .  .      :.    :  

MG06681.1 	PRS0---------~-------PTKDQRQAVNRFNRYVLGDAYVKELARLHPRSRE~DVKK
NCU01803.1	PRN~SCCPHYTLR~LDSTEFKPTKDQRQTVNRFNNYVIGPDYTHAAARVHPRTRQ~EARK
FG10878.1 	QRR~SCCPHYTIR~LDSTRFKPSRDQRQTINRFNKFVLGGLYTKEASRLYPRSRD~EAKK
AN6250.1  	LQR~SCCPHYTMR2LEASAYKPRRDQRKAINRWNKFVLGPEYIRRAAYLCPKTRE2EKKH
          	 :  :...  :    .::  .* :***:::**:*.:*:*  * :  : : *::*: : ::

MG06681.1 	RDTVFNLVERIHEAELDTLPSGPT--------PAHSFTVTLETDEFTEEK~YAVFENYQQ
NCU01803.1	RHNEFDLIDRIHEPEAQNLKAPPE--------PAHKFTVTLEPDEFTEEK~YLIFENYQR
FG10878.1 	RDNHFDLVERIHEAEDANVQKPPE--------AAHKLVVTLEHDDFTEEK~FAIYENYQR
AN6250.1  	RKCNFDLHDAVHAVEYSNVKRPTDPKTKRSLEPAHRFEVNLEGDTVSQAK2FDLFRKYQT
          	*.  *:* : :*  *  .:   . ..:. : ..** : *.** * .:: * : ::.:** 

MG06681.1 	VVHNEPSHRVSRTGFKRFLCNSPVQRTTVTTAEGYMRKLGSYHQCYRLDG~KLVAIGVLD
NCU01803.1	LVHKEGPDKISRHGFKRFLCNSPLRRETIYTSDGVQRQVGSFHQCYWLDG~KLVAIGVLD
FG10878.1 	VVHKDSPSEISKSGFKRFLCSSPLRRETMVVPDGRERRLGSYHQCYRLDG1---------
AN6250.1  	KVHKEDVSKWQTKDFKRFLCSG-LKRSEPNGRD-VQRRIGSWHQCYRLDG~KLIAVAVLD
          	 **::   . .  .******.. ::*      :   *::**:**** *** .  : .  .

MG06681.1 	LLPDCVSAVYFL~--YHESIHKHSPGKLGALREIAMAIEGGYRYWYSG~YYIHTCPKMQY
NCU01803.1	LLPHAVSSVYFV~--YHESFHSHNPGKLSAMREIALAREGGYRWWYPG~FYIHSCPKMKY
FG10878.1 	------------~-------------KLGALYEIALAIEEGYGWWYPG~FYIHSCSKMRY
AN6250.1  	LMPNGVSSVYIF2RSYDPDYEQWEIGKLSAMREIAFAVEQGYQYYYMG1YYIHSCQKMRY
          	  .   ::      :   .  . . .**.*: ***:* * ** ::* * :***:* **:*

MG06681.1 	KNDYSPQYILD~PQTLSWHHLGESEMRIFDSNGYARFSRLEQTSGEEKGT-----SADNK
NCU01803.1	KMDFKPQSVLD~PETLTWDLMDKEALAVFDAKHYVSLSRERQR--KESGD-----SPQ-I
FG10878.1 	KIDYSPQFVLD~PESLTWDPLDRGMLDLLDKKPFVSLSLEKKHAIKSDGEKPSVLNEEVK
AN6250.1  	KGNFRPQYILD1G----------ELTAKLNTRPYVSLSRDRATT-ATSGT----------
          	* :: ** :**                 :: . :. :*  .  :   .*           

MG06681.1 	IPEGDPRKDDEGDATTDEDDSMYEFLLRSDMPGIPTLEEMVSLDMENIAIAVNGYDQLFK
NCU01803.1	FEDHFIRPESDGEGDEDEGD----FMLGSSMPGIPSLEEMKQVDMDNLVLVSDAAPGYFL
FG10878.1 	TSDSTETKGPDSPGPGEEEDS--DWLFTSGMPGIPPIASAATWDMDHIALKIAPGGPLYE
AN6250.1  	--EANAADNAEAEAEINDEEVS---LFALHMPGVLTVDEVKKLDLGSWPLLIHG--SFVH
          	  :       :. .  :: :     ::   ***: .: .    *:    :          

MG06681.1 	AS~NLAVWAKQSV-LDFPGLKSKIAELVAAVGPDLMDKFCVDFRRRQPS-
NCU01803.1	AS~DLVSWEQQDIELDRKDIKSTIAELVAAMGTDVMGEICVDFRRRTRAL
FG10878.1 	TS~DLVSWDERGV-KEHPGLKAAVAELVAATGPDLMDRICVDFAPRR---
AN6250.1  	MS0DLVGWEKMPI-DEPQSIKGIVAELAATLGPTVMKDSAVVLFD-----
          	 * :*. * :  :  :  .:*. :***.*: *. :*   .* :
```
